# 3D printed tooth for endodontic training in dental education

**DOI:** 10.1038/s41598-025-06081-y

**Published:** 2025-06-20

**Authors:** Isabella Di Lorenzo, Michael del Hougne, Gabriel Krastl, Marc Schmitter, Christian Höhne

**Affiliations:** 1 Department of Prosthodontics, University of Würzburg, Pleicherwall 2, 97070 Würzburg, Germany; 2 Department of Conservative Dentistry and Periodontology, University of Würzburg, Pleicherwall 2, 97070 Würzburg, Germany

**Keywords:** Dental education, 3D printing, Printed tooth, RCT, Endodontic treatment, Additive manufacturing, Endodontics, Dental education, Dental clinical teaching, Endodontic files, Root canal treatment

## Abstract

In dental education root canal treatment is primarily trained with natural teeth models or transparent acrylic blocks. A newly constructed, 3D printed practice tooth can be produced cost-efficiently and reproducibly while based on a natural root canal anatomy. Based on a micro-CT scan of an extracted tooth number 36, a dataset was reconstructed (Autodesk Inventor 2019, Autodesk Inc.) and subsequently 3D printed (Form 3B, Formlabs Inc.). In a hands-on course, dental students performed root canal treatments on 3D printed practice teeth. The practice teeth were prepared and filled. A reciprocating system (Reciproc, VDW GmbH) and a rotating preparation system (Pro Taper Next, Dentsply Sirona GmbH) were utilized. Students completed a questionnaire to evaluate practice materials, learning success, and learning process. The students rated suitability as a practice option (*p* < 0.001) and handling (*p* = 0.001) of the 3D printed practice tooth, as well as natural tooth models, significantly better than acrylic blocks.

**Supplementary Information:**

The online version contains supplementary material available at 10.1038/s41598-025-06081-y.

## Introduction

Transparent acrylic blocks are mainly utilized for practicing root canal treatments, with single-rooted (Flex Master practice block, V040245, VDW, Munich, Germany) and two-rooted (root canal study model, S1-U4, J. Morita, Tokyo, Japan) blocks. These blocks only represent a root canal system without tooth crown.

Extracted natural teeth are applied for practice, as well, and can be anchored in a dental simulation unit. The use of natural teeth is of particular relevance in endodontic education due to their anatomical and characteristic features. Natural tooth models are inexpensive to produce, however, they pose hygienic and ethical challenges. The advantages and disadvantages of natural teeth compared to printed practice teeth have already been evaluated^1^.

Their utilization has been criticized by some universities’ ethics committees, and the regulations for their use have been tightened. In addition to patient consent, further documentation of consent for university use is required. This increase in administrative effort could reduce dentists’ willingness to collect extracted teeth^2,3^. The preparation and disinfection of the teeth are problematic, and the properties of the natural teeth change due to the preparation process^4,5^. Furthermore, generating a sufficient number of natural teeth with adequate quality represents a challenge^6^. As the anatomy of individual root canals is not identical and can differ significantly. This results in uneven learning outcomes, thus creating unfair conditions. In contrast, the 3D printed practice tooth offers identical conditions for each tooth and does not pose any hygienic or ethical-legal issues.

The aim of the study was to test and evaluate this practice option for learning root canal treatments in student education.

The null hypothesis stated that there would be no difference in students’ evaluations between the existing raining methods (i.e., transparent acrylic blocks and natural teeth models) and the 3D-printed practice teeth.

## Materials and methods

Voluntary participation of participating students in the hands-on course and completion of the questionnaire were prerequisites. The study was approved by the Institutional Review Board (University of Würzburg, Germany)—the collected data had to be fully and irreversibly anonymized (20181116 01) and the use of anonymized existing scans and radiological data for the use of printed teeth in education was granted (20210823 02). All methods were carried out in accordance with relevant guidelines and regulations and experimental protocols were approved by the Institutional Review Board (University of Würzburg, Germany). An informed consent was obtained from all subjects and/or their legal guardian(s).

### Design of 3D printed practice tooth and practice materials

**Fig. 1:**
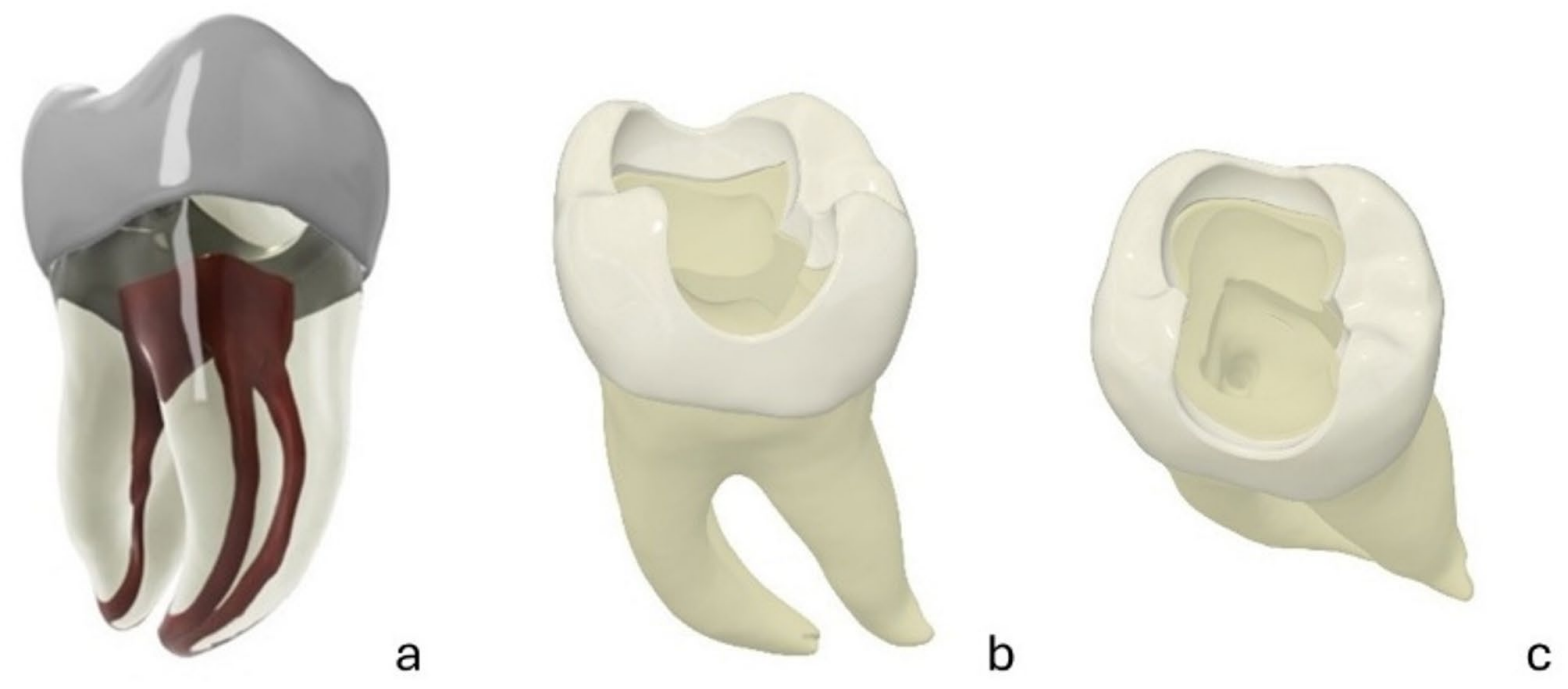
Anatomy of the practice tooth, schematic illustration. (**A**) Transparent visualization of the root canal system. (**B**) Lateral view. (**C**) Occlusal view with access cavity (Source: own figure).

An extracted left mandibular molar was voluntarily provided by a donor for study purposes and utilized in this study. Data was anonymized such that no references to the donor were possible. First, a micro-computed tomography of the extracted tooth was performed by the Fraunhofer Institute for Integrated Circuits (MetRIC Micro and Region of Interest, Fraunhofer IIS, Erlangen, Germany). Using Autodesk Inventor 2019 (Autodesk Inc., San Rafael, California, USA), the micro-computed tomography data was reconstructed and modified, as shown in Fig. 1. The modification involved refining the data set to ensure that the relevant structures were clearly identifiable and creating the access cavity. No other anatomical features were altered. This enabled a practice tooth with a realistic root canal anatomy and an access cavity.

**Fig. 2:**
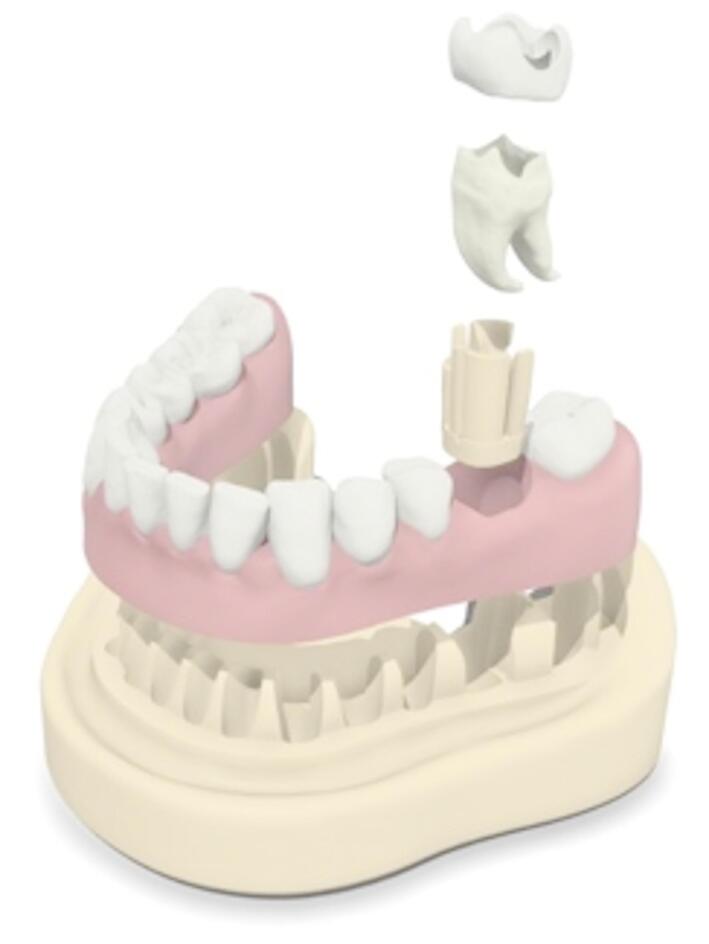
3D printed model, schematic illustration. From basal: model, gingival mask, holder for endodontic tooth, root and crown dentin, enamel cap (Source: own figure).

A mandibular model, previously utilized for educational training of caries excavation^1,7^was modified for this study, as illustrated in Fig. 2. It was anchored in a phantom head and served to fix the practice tooth, creating a patient-like situation.

### Additive manufacturing of practice materials

The additive manufacturing of the practice materials was conducted with a 3D printer employing the stereolithography (SLA) process, where a photosensitive resin is selectively hardened into a solid using laser technology. Form 2 (Formlabs GmbH 2024) and Form 3B (Formlabs GmbH 2024) were utilized. Utilization of two different printers was due to local resource availability and allocation and not based on the 3D printers’ specifications. The Form 2 has a 140 μm laser spot size, while the Form 3B has 85 μm laser spot size and an XY resolution of 25 μm. Both printers enable a layer thickness of up to 25 μm, depending on the resin.

Items were prepared for 3D printing using the software PreForm 3.0.1 (Formlabs Inc., Somerville, Massachusetts, USA). As the resins vary in their material properties, i.e. hardness, color, and surface texture, different materials were used for the individual components of the practice teeth.

The dentin portions were printed using a Form 2 printer with White Resin V4 and Model Resin V2 (Formlabs Inc.) at a layer thickness of 50 μm. The enamel portions were printed with a Form 3B printer using a white, glass-filled resin (Rigid 4000 Resin, Formlabs Inc.), also at a 50 μm layer thickness. The latter had increased strength values and a smooth and white surface yielding in optical similarities to natural enamel. The tooth root with the crown dentin were printed separately from the enamel cap as different materials were utilized, as shown in Fig. 2.

Post-Processing and Finishing of Practice Materials.

The post-processing of the two materials followed the manufacturer’s instructions. It included washing the objects with 100% isopropanol to remove any liquid resin, airdrying and hardening. For the dentin parts, the root canals were additionally rinsed with 100% isopropanol for 60 s per canal, using disposable syringes equipped with endodontic irrigation tips. The patency of the root canals was assessed after completion of the entire post-processing procedure using an ISO size 8 root canal file. In cases of blockage, the respective tooth was excluded from the study.

**Fig. 3:**
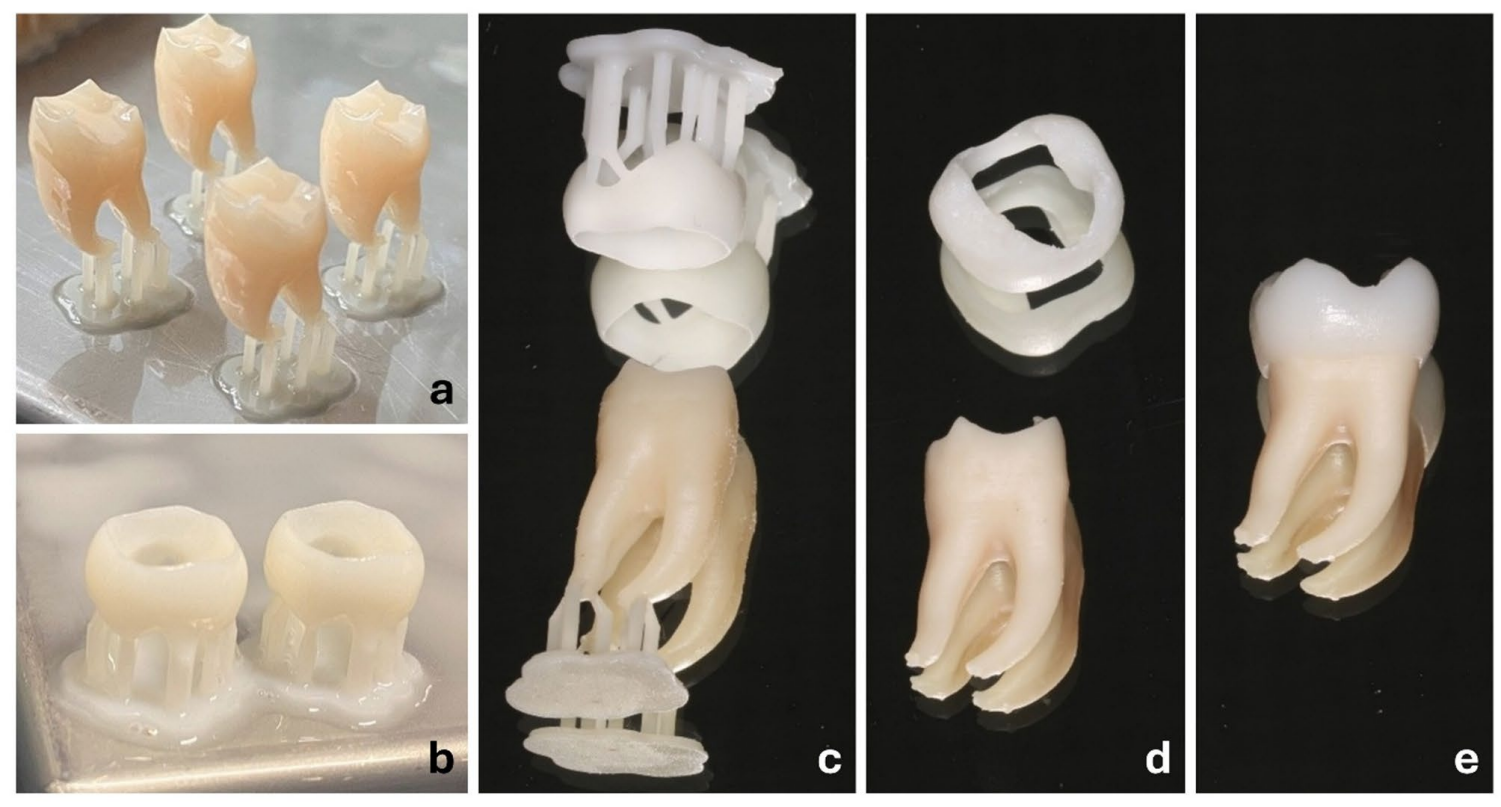
3D printed practice tooth model production process. (**a**) Dentin parts with resin residues on build platform. (**b**) Enamel parts with resin residues on build platform. (**c**) Washed objects with support structures. (**d**) Enamel and dentin parts prepared for assembly. (**e**) Assembled tooth (Source: own figure).

The assembly included bonding the enamel cap to the dentin. Therefore, model resin (Model Resin V2, Formlabs Inc.) was first applied to the inside of the enamel cap with a disposable micro applicator and then placed on the dentin part. Excess resin was removed, and the bonding area was light-cured. Additionally, the enamel cap was coated with a thin layer of a light-curing one-component varnish, Plaquit (Dreve Dentamid GmbH, Unna, Germany) and light-cured, as well. The varnish layer gave the enamel cap a shiny surface, resulting in a more natural-looking final product, as illustrated in Fig. 3.

### Hands-on course

Fifth-year students participated in a voluntary hands-on at the Department of Dental Prosthetics, University Hospital of Würzburg.

Students had already gained 1.5 years of clinical experience in performing root canal treatments. In their previous training courses, students had practiced root canal preparation and filling on transparent acrylic training blocks and natural tooth models. Thus, the students had experience handling acrylic training blocks (various models with one or two root canals) as well as natural teeth, including incisors, premolars, and molars.

The hands-on course was held on two occasions, separated by 14 days and each student processed a total of three practice teeth, ensuring that both reciprocating and rotary preparation systems were utilized. For the reciprocating system, Reciproc Blue R25 files (Reciproc, VDW GmbH) were used. The rotary system involved a sequence of three files, X1 to X3, from the ProTaper Next system (ProTaper Next, Dentsply Sirona GmbH). Root canal preparation was performed using the X-Smart Plus endodontic motor (Dentsply Sirona GmbH), which proved particularly well-suited for the hands-on training due to its compatibility with both rotary and reciprocating instrumentation techniques.

The students were already familiar with the Reciproc system from their previous coursework. At the beginning of the first session, they received an introduction to the upcoming tasks through a presentation of approximately 15 min. During this presentation, the preparation systems were introduced and explained, providing participants with a theoretical foundation before the practical training.

**Fig. 4:**
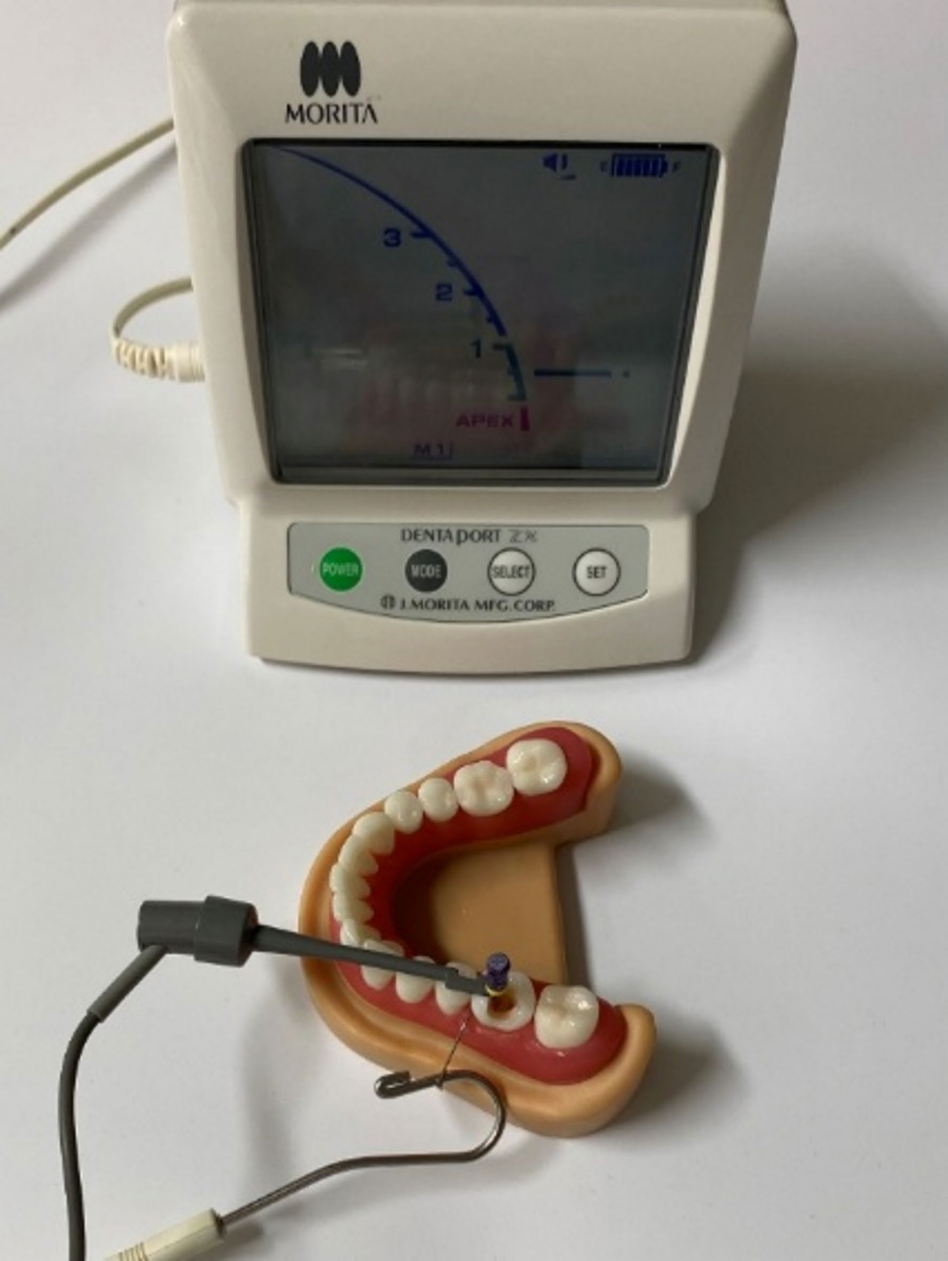
Determination of length of root canals (Source: own figure).

Additionally, a model was provided for determination of the length of the root canals. An electronic apex locator (DentaPort Root ZX, J. Morita Europe GmbH) and a hand-bent wire element were utilized with ultrasound gel (Ultraschall Gel Clear, Konix, Istanbul, Turkey) in the root canal system and apex region for enabling electrical conductivity, as shown in Fig. 4. This setup enabled precise measurement of root canal lengths and more closely simulated clinical conditions than conventional training methods.

### Questionnaire

After the practical exercises were completed, each student voluntarily completed a questionnaire (see Table 1). The Institute for Medical Teaching and Training Research (IMLA) of the University of Würzburg collaborated for the design of the questionnaire. It was digitized with EvaSys (Electric Paper Evaluation System, evasys GmbH, 2023) and consisted of both free-text questions and predefined answer options that students could evaluate using a visual analog scale (VAS). The VAS had a 10 cm stretch without scaling and opposing statements at each end of the stretch.

The questionnaire was divided into different sections. An introduction section was followed by a brief section regarding personal data. The properties of the printed teeth were compared with natural teeth, as well as other training options. The preparation methods were evaluated and learning outcomes and process were self-assessed. Free text questions solicited suggestions for improvements and the advantages of the printed teeth.

### Statistics

Data processing was performed using Excel (Version 2301, Microsoft 365 Apps for Enterprise, Microsoft Corporation, 2023). IBM SPSS Statistics (Version 29.0.0.0, IBM Deutschland GmbH, 2023) and G*Power (Version 3.1.9.7) were used for statistical analysis. A descriptive analysis was conducted. Data was tested for normal distribution using the Shapiro–Wilk test. Statistical significance was assessed by the Mann–Whitney U test. When comparing more than two independent groups, the non-parametric Kruskal–Wallis test was applied, followed by Dunn’s test with Bonferroni correction. The responses to the free text questions were manually grouped by two independent examiners, who analyzed them for common themes and categorized them accordingly. In cases of disagreement, the examiners engaged in a thorough discussion to reach a consensus, ensuring consistent and reliable categorization of the data. This collaborative approach helped maintain the validity of the grouping process despite the absence of a formal inter-rater reliability measure. Internal consistency of the questionnaire was evaluated by computing Cronbach’s alpha. The significance level was set at α = 0.05.

**Table 1:** Questionnaire for hands-on course.

2. Personal data
2.2 Please enter your age
2.3 Have you completed an education in the dental sector prior to studying dentistry?
2.4 If yes, which one?
2.5 Please enter your gender
2.6 Which file system did you start with in the first session
2.7 The work in the preclinical course of restorative dentistry, especially the exercises in root canal treatment, came easily to me
2.8 The patient treatment in the clinical courses was easy for me
2.9 I take great pleasure in the practical aspects of studying dentistry
2.10 This is how I assess my manual skills.
2.11 During the preclinical and clinical courses, I had plenty of opportunities to practice root canal treatment.
3. Properties of printed teeth in comparison to natural teeth
3.1 Realistic feeling when determining the length of the root canal
3.2 Realism of the printed teeth
3.3 Realistic feeling during root canal preparation
3.4 Realistic feeling during root canal filling
3.5 Realistic root canal anatomy
3.6 Realistic pulp cavity
4. Characteristics of printed teeth in comparison with other training options
4.1 Suitability as a training option of printed tooth
4.2 Suitability as a training option of acrylic resin block
4.3 Suitability as a training option of natural teeth model
4.4 Handling of printed tooth
4.4 Handling of acrylic resin block
4.5 Handling of natural teeth model
5. Characteristics of preparation methods
5.1 Handling of Reciproc
5.2 Handling of ProTaper Next
5.3 I would utilize the Reciproc system myself
5.4 I would utilize the ProTaper Next system myself
6. Assessment of learning outcome
6.1 Subjective learning outcome with Reciproc
6.2 Subjective learning outcome with ProTaper Next
6.3 After this hands-on course I feel well prepared for root canal treatments
7. Assessment of learning process
7.1 The hands-on has sparked my enthusiasm to improve my skills in root canal preparation
7.2 For my studies I desire more exercises with 3D printed teeth
7.3 I can imagine the entire training in preparation for patient treatment being conducted solely with printed teeth
8. Free-text questions
8.1 What could be improved on the printed teeth?
8.2 Which advantages do printed teeth offer in dental education, in your opinion?

## Results

**Fig. 5:**
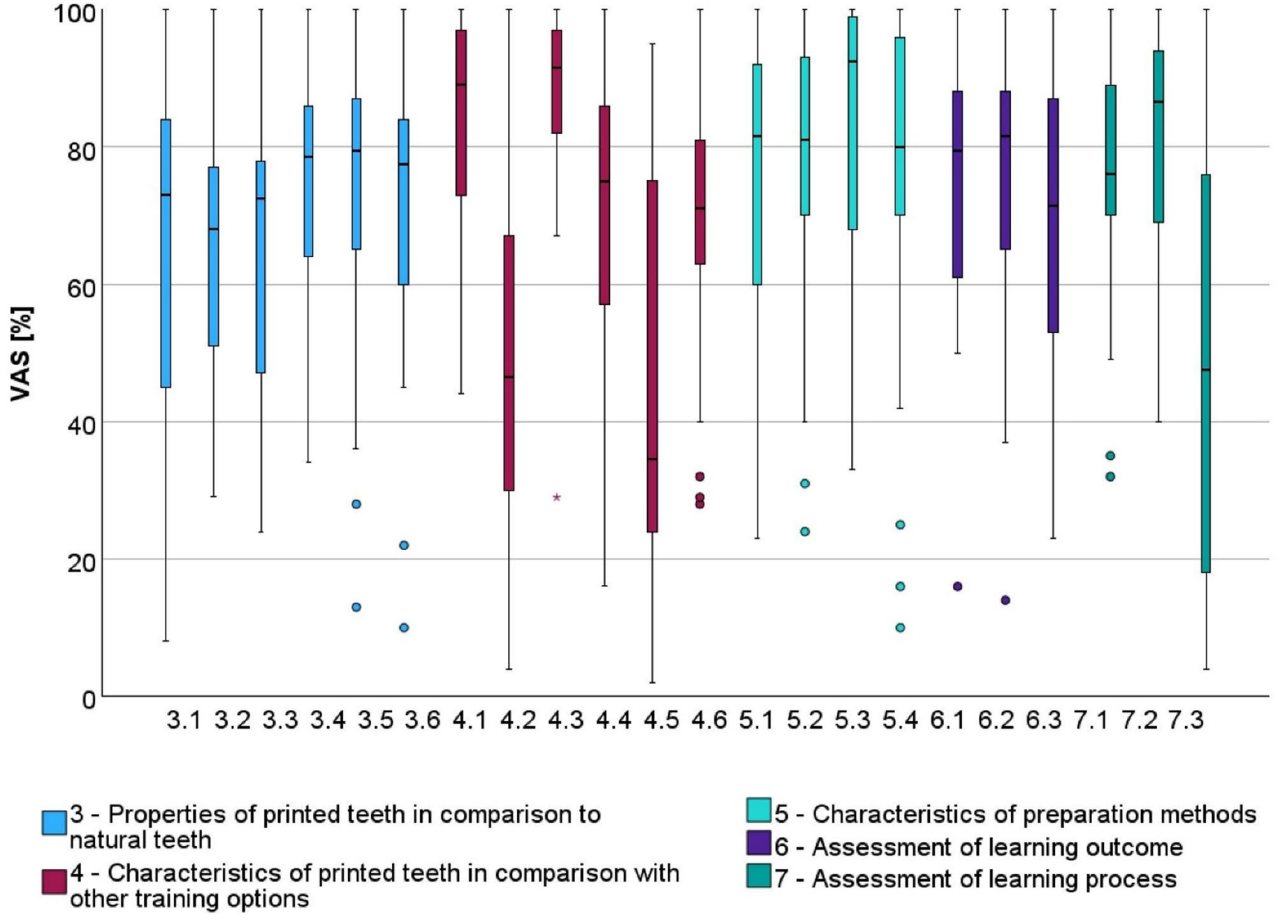
Boxplot with results from questionnaire (VAS items 3.1 to 7.3) (Source: own figure).

Overall, 38 students completed the questionnaire—results can be found in Table S1. The completed questionnaires were manually entered into an Excel spreadsheet and the visual analog scale was read with a digital caliper and resulting percentages calculated. Results from 3.1 to 7.3 are visualized in a boxplot in Fig. 5.

The 38 students were composed of 29 women and 9 men. They had an average age of 24.9 (± 3.1) years. 6 students had completed an education prior to studying dentistry as dental technicians (*n* = 3) and medical and dental assistants (*n* = 3). The students rated their ease with previous exercises regarding endodontic treatments without patients at Ø 61.1% (± 21.7), and the patient treatment at the clinical courses at Ø 60.8% (± 18.1). Students enjoyed practical elements of their education at Ø 79.4% (± 16.7) and self-evaluated their manual skills at Ø 65.0% (± 12.4). Sufficient training opportunities for endodontic treatments within the preclinical and clinical courses were rated at Ø 57.0% (± 27.3).

When evaluating the printed training teeth in comparison to natural teeth, the length measurement was rated Ø 64.9% (± 25.4) and the realism of the printed teeth at Ø 65.5% (± 16.8). The realistic feel during root canal preparation was rated at Ø 64.7% (± 20.8), during root canal filling at Ø 74.4% (± 15.9), realistic canal anatomy at Ø 73.9% (± 19.4), and realistic pulp chamber at Ø 71.6% (± 20.0).

Suitability for practice was rated for printed tooth (Ø 84.0% ±15.4) significantly better (*p* < 0.001) compared to acrylic resin blocks (Ø 49.1% ±25.0), and the acrylic resin blocks as significantly less suitable (*p* < 0.001) compared to natural tooth models (Ø 88.2% ±13.1). Thus, compared to the natural tooth models, students rated the suitability of the printed tooth as a practice option slightly lower.

The handling of the printed teeth (Ø 70.3% ±21.4) and the natural tooth models (Ø 70.9% ±19.3) were rated similarly, while acrylic resin blocks (Ø 46.4% ±28.5) were considered to have a more difficult handling. Students rated the handling of acrylic resin blocks as significantly more difficult compared to printed teeth (*p* = 0.001) and natural tooth models (*p* = 0.001).

Regarding the preparation methods, the handling of the Reciproc system (Ø 74.6% ±20.9) was rated as more difficult compared to ProTaper Next (Ø 78.1 ± 18.1). However, when asked which system students preferred to use themselves, Reciproc (Ø 82.6 ± 20.0) was rated higher than ProTaper Next (Ø 76.0 ± 23.1).

The subjective learning success was rated slightly higher for ProTaper Next (Ø 75.5 ± 18.9) compared to Reciproc (Ø 75.3 ± 18.3). Additionally, the students indicated that they felt well-prepared for root canal preparation due to the hands-on course (Ø 69.1 ± 18.3).

Regarding the learning process, students rated at Ø 75.9% (± 17.1) that the hands-on course had sparked their enthusiasm to improve their root canal preparation skills. The question of whether more exercises with 3D printed practice teeth were desired in their education was rated at Ø 81.5% (± 16.1). However, the completion of the entire training for patient treatment with 3D printed practice teeth was rated at only Ø 48.6% (± 30.9).

The reliability analysis was determined for VAS items 2.7 to 7.3 with Cronbach’s alpha, yielding a value of ∝ = 0.814. This can be considered as very high or excellent.

The responses for the free-text questions were grouped and similar answers were categorized and tallied.

The following suggestions for improvement of the 3D printed practice teeth were made:

- Improvement of the tooth’s retention in the model (*n* = 30).
- Optimization of the material hardness (*n* = 7).
- Closing the access cavity, to integrate access cavity preparation into the practice (*n* = 5).
- Reducing the size of the access cavity (*n* = 2).
- Generating variations of root canal anatomies (*n* = 3).
- Making the pulp chamber darker (*n* = 1).
- Simulation of denticles (*n* = 1).
- Providing the option to take length measurement radiographs (*n* = 1).
- Enabling self-determination of working length (*n* = 1).
- Optimizing the curvature of the canals (*n* = 1).

The following advantages of the 3D printed practice tooth for education were stated:

- Realistic representation (*n* = 24).
- Good practice opportunity in preparation of patient treatments (*n* = 10).
- Ability to practice with alternating root canal anatomies (*n* = 7).
- Less preparation efforts required compared to natural tooth models (*n* = 4).
- Cost-effective production (*n* = 4).
- Easier to perform root canal treatment compared to natural tooth models (*n* = 3).
- Opportunity to practice with several teeth (*n* = 3).
- Conditions are fairer due to the same level of difficulty (*n* = 3).
- No risk to patients (*n* = 1).
- Teeth can easily be replaced (*n* = 1).
- Practice on a phantom head is possible (*n* = 1).

## Discussion

The questionnaire results highlight the potential of the printed teeth and confirm their added value in dental education.

3D printing, an additive production process, was elemental to producing the teeth, as it enables the fabrication of cavities and root canals—this is not possible by subtractive production, such as milling. The high precision of Form 2 and Form 3B enabled the production of the teeth with filigree root canals. 3D printing of teeth provided a cost-effective production^8^. While 3D printing is an established method for producing training utensils, it also has the potential to manufacture dental prostheses for patient use when an appropriate resin is utilized^9^. Reymus et al. printed teeth for endodontic treatment training with a Form 2 and revealed a trueness ranging from 50.9 μm to 104.3 μm and a precision ranging from 43.5 μm to 68.2 μm. Overall, 3D printed teeth are suitable for production of teeth for endodontic treatment training in dental education^4^. As sufficient training opportunities for endodontic treatments within the preclinical and clinical courses were rated at Ø 57.0% (± 27.3), it is emphasized that more training is required, especially as the ease of endodontic treatments were rated at Ø 61.1% (± 21.7) without patients and at Ø 60.8% (± 18.1) in the clinical courses. Overall, the 3D printed practice tooth was rated significantly better than acrylic resin blocks regarding suitability for practice (*p* < 0.001) and handling (*p* = 0.001). This was enhanced by positive evaluations of realism of the printed teeth, feel during root canal preparation and filling, canal anatomy and pulp chamber. However, the suitability of the printed teeth as a practice option and handling were rated slightly lower than natural teeth, pointing out the limitations of simulation of natural teeth. This was also attested by students as a completion of the entire training for patient treatment with 3D printed practice teeth was rated at only Ø 48.6% (± 30.9). Both preparation methods were evaluated similar at handling, however, students favored Reciproc. Providing varying systems enhances students’ experiences with root canal treatment. Similar subjective learning successes were attested and overall, students felt well-prepared for root canal preparation due to the hands-on course (Ø 69.1 ± 18.3). The hands-on course sparked students’ enthusiasm and there was great desire for further exercises with 3D printed practice teeth. Students valued the realistic representation (*n* = 24) and the good practice opportunity in preparation of patient treatments (*n* = 10). The method of this study required less preparation effort compared to natural tooth models (*n* = 4) and overall, a cost-effective production (*n* = 4). Each tooth required 0.97 ml of resin at a cost of 0.17€ per unit. The printing process took 4 h and 12 min for the dentin parts and 5 h and 26 min for the enamel parts. However, improvements suggested by the students included an improvement of the tooth’s retention in the model (*n* = 30), which could be obtained by optimizing spacer between tooth and model or implementation of a retention element such as a screw. Although the access cavity was provided on purpose, aiding fair conditions and equal levels of difficulty (*n* = 3), students desired closing the access cavity (*n* = 5) or reducing its size (*n* = 2) to enhance the exercise. The findings of the questionnaire allowed to confidently rejecting the null hypothesis, as students evaluated the 3D printed practice teeth superior to transparent acrylic blocks and equal to natural teeth models. The type of questionnaire utilized in this study represents an established method which can be found in other studies^7,10^. The VAS provide an objective and reliable instrument with high validity^11^. In this study, 38 students participated in the hands-on course and completed the questionnaire. Therefore, the findings cannot be generalized. However, although the production costs are limited, the manual assembly was time-consuming and, in the context of hands-on courses, proved to be a labor-intensive process^10,12^.

The material properties of the printable resins represent a limiting factor. Enamel is the hardest material produced by the body and therefore difficult to imitate^13^. Others concluded that students preferred extracted human teeth to 3D-printed teeth due to physical characteristics in endodontic training^14^. Other studies also recognized the benefits of artificial resin due to its multiple advantages but rejected it as a replacement for natural teeth^15,16^.

A key strength of providing a model for root canal length determination lies in its ability to accommodate customized root canal anatomies. This enables simulation of a wide variety of anatomical variations and complexities encountered in clinical practice. By incorporating adaptable and realistic root canal configurations, the model broadens students’ exposure to diverse endodontic scenarios, enhancing their diagnostic and procedural skills. This customization not only personalizes the learning experience but also better prepares students for real-world challenges by providing a more comprehensive and varied training environment.

Overall, the 3D printed practice teeth for training of root canal treatments revealed benefits for education.

## Conclusion

The 3D printed practice teeth allowed students to practice root canal treatment independently and with a targeted approach. They serve as a valuable alternative to existing training methods and could be further improved with future advancements. Overall, this realistic training method provides a fair and cost-effective learning environment without hygienic or ethical concerns.

## Electronic supplementary material

Below is the link to the electronic supplementary material.

Supplementary Material 1

## Data Availability

All data needed to evaluate the conclusions in the paper are present in the paper.
