# Supplementary material for: 3D printed tooth for endodontic training in dental education
Source: Sci Rep. 2025 Jun 20;15:20185. doi: 10.1038/s41598-025-06081-y (PMC12181308; doi:10.1038/s41598-025-06081-y)
Supplement: Supplementary file 1 — Supplementary Material 1 [file 41598_2025_6081_MOESM1_ESM.docx]

# Supplementary Tables

|  | **Minimum** | **Maximum** | **Mean** | **Standard  Deviation** |
| --- | --- | --- | --- | --- |
| 2.2 | 22 | 35 | 24.9 | 3.1 |
| 2.7 | 15 | 100 | 61.1 | 22.0 |
| 2.8 | 12 | 100 | 60.8 | 18.4 |
| 2.9 | 38 | 100 | 79.4 | 16.9 |
| 2.10 | 47 | 100 | 65.0 | 12.5 |
| 2.11 | 2 | 100 | 57.0 | 27.7 |
| 3.1 | 8 | 100 | 64.9 | 25.7 |
| 3.2 | 29 | 100 | 65.5 | 17.0 |
| 3.3 | 24 | 100 | 64.7 | 21.1 |
| 3.4 | 34 | 100 | 74.4 | 16.1 |
| 3.5 | 13 | 100 | 73.9 | 20.1 |
| 3.6 | 10 | 100 | 71.6 | 20.3 |
| 4.1 | 44 | 100 | 84.0 | 15.4 |
| 4.2 | 4 | 100 | 49.1 | 25.3 |
| 4.3 | 29 | 100 | 88.2 | 13.3 |
| 4.4 | 16 | 100 | 70.3 | 21.4 |
| 4.5 | 2 | 95 | 46.4 | 28.5 |
| 4.6 | 28 | 100 | 70.9 | 19.3 |
| 5.1 | 23 | 100 | 74.6 | 21.2 |
| 5.2 | 24 | 100 | 78.1 | 18.3 |
| 5.3 | 33 | 100 | 82.6 | 20.0 |
| 5.4 | 10 | 100 | 76.0 | 23.1 |
| 6.1 | 16 | 100 | 75.3 | 18.5 |
| 6.2 | 14 | 100 | 75.5 | 19.1 |
| 6.3 | 23 | 100 | 69.1 | 22.1 |
| 7.1 | 32 | 100 | 75.9 | 17.1 |
| 7.2 | 40 | 100 | 81.5 | 16.1 |
| 7.3 | 4 | 100 | 48.6 | 30.9 |
|  | **Responses with count** | | | |
| 2.3 | - no (n=32) - yes (n=6) | | | |
| 2.4 | - dental technician (n=3) - medical or dental assistant (n=3) | | | |
| 2.5 | - male (n=9) - female (n=29) | | | |
| 8.1 | - Improvement of the tooth's retention in the model (n=30) - Optimization of the material hardness (n=7) - Closing the access cavity, to integrate access cavity preparation into the practice (n=5) - Reducing the size of the access cavity (n=2) - Generating variations of root canal anatomies (n=3) - Making the pulp chamber darker (n=1) - Simulation of denticles (n=1) - Providing the option to take length measurement radiographs (n=1) - Enabling self-determination of working length (n=1) - Optimizing the curvature of the canals (n=1) | | | |
| 8.2 | - Realistic representation (n=24) - Good practice opportunity in preparation of patient treatments (n=10) - Ability to practice with alternating root canal anatomies (n=7) - Less preparation efforts required compared to natural tooth models (n=4) - Cost-effective production (n=4) - Easier to perform root canal treatment compared to natural tooth models (n=3) - Opportunity to practice with several teeth (n=3) - Conditions are fairer due to the same level of difficulty (n=3) - No risk to patients (n=1) - Teeth can easily be replaced (n=1) - Practice on a phantom head is possible (n=1) | | | |

***Table S1****: Results of the questionnaire.*
